# Design of a novel multifunction decision support/alerting system for in-patient acute care, ICU and floor (AlertWatch AC)

**DOI:** 10.1186/s12871-021-01411-9

**Published:** 2021-07-24

**Authors:** Douglas A. Colquhoun, Ryan P. Davis, Theodore T. Tremper, Jenny J. Mace, Jan M. Gombert, William D. Sheldon, Joseph J. Connolly, Justin F. Adams, Kevin K. Tremper

**Affiliations:** 1 Department of Anesthesiology, University of Michigan, 1500 E. Medical Center Drive, Ann Arbor, MI 48109 USA; 2 AlertWatch Headquarters, 330 E. Liberty Street, Ann Arbor, MI 48104 USA

**Keywords:** Monitoring, Surveillance, Acute Care, ICU, Decision Support, EHR, Inpatient Medicine

## Abstract

**Background:**

Multifunction surveillance alerting systems have been found to be beneficial for the operating room and labor and delivery. This paper describes a similar system developed for in-hospital acute care environments, AlertWatch Acute Care (AWAC).

**Results:**

A decision support surveillance system has been developed which extracts comprehensive electronic health record (EHR) data including live data from physiologic monitors and ventilators and incorporates them into an integrated organ icon-based patient display. Live data retrieved from the hospitals network are processed by presenting scrolling median values to reduce artifacts. A total of 48 possible alerts are generated covering a broad range of critical patient care concerns. Notification is achieved by paging or texting the appropriated member of the critical care team. Alerts range from simple out of range values to more complex programing of impending Ventilator Associated Events, SOFA, qSOFA, SIRS scores and process of care reminders for the management of glucose and sepsis. As with similar systems developed for the operating room and labor and delivery, there are green, yellow, and red configurable ranges for all parameters. A census view allows surveillance of an entire unit with flashing or text to voice alerting and enables detailed information by windowing into an individual patient view including live physiologic waveforms. The system runs via web interface on desktop as well as mobile devices, with iOS native app available, for ease of communication from any location. The goal is to improve safety and adherence to standard management protocols.

**Conclusions:**

AWAC is designed to provide a high level surveillance view for multi-bed hospital units with varying acuity from standard floor patients to complex ICU care. Alerts are generated by algorithms running in the background and automatically notify the selected member of the patients care team. Its value has been demonstrated for low acuity patients, further study is required to determine its effectiveness in high acuity patients.

## Background

Implementation of electronic health record (EHR) systems and ubiquitous presence of monitoring devices in acute care environments have led to inpatient care being associated with large streams of continuously updating information. These electronic systems have the advantage of providing comprehensive patient data anywhere at any time, which can be used to improve care by keeping providers appraised of patient’s status. All inpatients require active management to insure safe, effective and efficient care. The critical care and monitored environments uses continuous physiologic and device monitoring with parameter specific incorporated alarms to improve safety. Unfortunately, this large volume of continuous data may produce an overwhelming number of alarms or alerts, with the potential to harm due to alarm fatigue [1–3].

Integrating these multiple streams of rapidly changing data into a system which can prioritize and display a large amount of data in an easily understood manner, may help address this issue. This could be considered analogous to the development of the multifunction flight displays in the modern aircraft that take data previously represented across many dials or indicators and present a single reference screen and includes prioritized alerts to pilots [4]. When considered across multiple patients it is necessary to have a readily prioritized and easy to understand way of reviewing large amounts of clinical data to enable identification of patients in most need of immediate attention [4]. This may be considered analogous to a “flight control tower”.

AlertWatch, Inc. has developed software which provides live, real-time decision support alerts [5, 6]. The displays synthesize multiple laboratory, history and physiologic parameters into an intuitive icon-based display for easy identification of organ system problems and alert when systems/parameters are out of pre-specified, patient specific customizable ranges [5, 6]. The system also allows delivery of text alerts to devices (pagers, phones etc.) carried by the care team, in a role specific manner to designated individuals. These systems have been developed and implemented for Operating Room (AWOR) and for Labor & Delivery (AWOB) care areas [5–9]. Recently, Safavi et al. reported on a simple version of AlertWatch developed for remote monitoring of patients on a surgical floor unit [10]. In this 17 month study they found that 88% of the alerts were actionable and concluded that they were unlikely to cause alarm fatigue [10]. This current manuscript describes a more complex version of AlertWatch which has been developed for critical care and floor patients, AlertWatch Acute Care (AWAC). Remote access to patient status and expanded monitoring has become of even more importance given the necessity of protecting care staff by limiting unnecessary close patient contact during pandemic situations. The purpose of this paper is to describe this system with its tiered organ system-based alerts and the logic used in their determination.

### Implementation

Alertwatch uses a Microsoft (Redmond, WA) stack for data collection and analysis. Data are pulled via web services or received via feeds like HL7, standardized and then analyzed for notification and display. The front end uses javascript and is written using Vue.js. AWAC implementation is installed on a secured server provided by the institution. The variables are mapped from the EMR and the alert configuration is based on institutional preferences.

## Results

### System description

AWAC is comprised of a census view and a patient view with decision support alerts which are designed based on hospital protocols and current literature.

### Data acquisition

Data are acquired from multiple sources in the EMR: laboratory, nursing flow sheet, physiologic network for live monitored data, device data (ventilator, mechanical circulatory support, ECMO & LVAD), patient demographics, patient diagnosis and co-morbidities by the International Classification of Disease 10^th^ edition (ICD 10) codes, medications and patients care team contact information. The nursing flow sheet contains management data, some of which are displayed and also used in the calculations. For example, urine output is displayed, graphed and used in calculation of a rate in ml/kg/hr.

Similarly, documented chest tube output is displayed and also used for an alert when the rate exceeds a designated amount, eg greater than 300 ml in one hr. Finally, medication administration data can be used in alerting. When insulin is administrated a time is set for one hr to check for a glucose measurement. If not seen in the lab extract, an alert is sent to consider rechecking glucose. A complete list of alerts and limits are presented in Tables 1 (presented at the end of the paper) and 2 below.

**Table 1:**
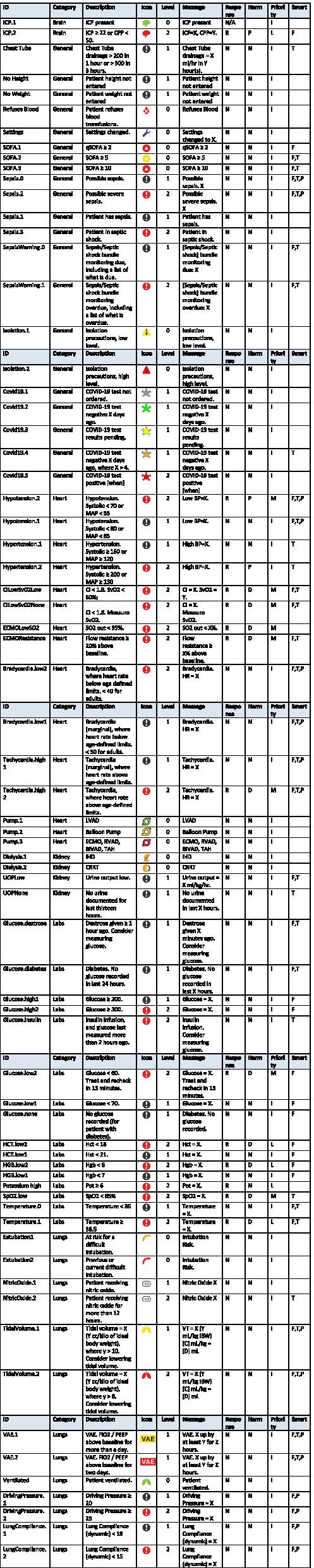
Icon definitions and the alerts associated with many of the icons

The numerical alert triggers can be configured upon institutional installation. Individual alert limits may be changed; note the wrench icon for setting changes. The alerts, associated with calculations such as SOFA scores and bradycardia limits, have literature references for those calculations

**Table 2:**
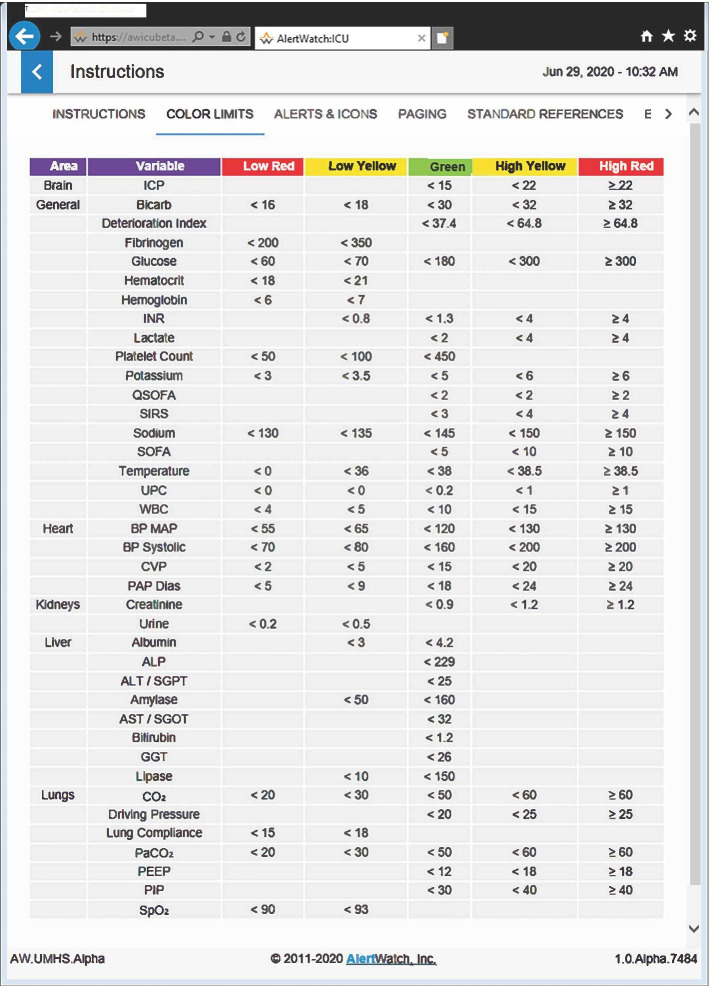
Color Codes for Alert Limits

These color limits are configurable on institutional installation

### Alerts and icons

AWAC has a series of alerts and icons. The alerts range from simple high/low thresholds to more complex alerts involving calculations and several types of data extracted from the EMR. The alerts are also presented in a severity hierarchy following the International Organization for Standardization (ISO) recommendations for Health Informatics, Table 1 [11]. AWAC also has a series of icons representing important aspects of the patients care to enable easy identification on the Census View, described below. Examples icons are; a lung for mechanical ventilation, a pump for mechanical circulatory support (ECMO), a brain for ICP monitoring and so on. Icons are color coded based of severity. These alerts and icons are described in detail, Table 1.

### Alerts

Certain alerts, typically called smart or intelligent alerts, have additional logic to help improve their relevancy and accuracy and to reduce alarm fatigue. If the field is empty, the alert activates with a simple threshold or limit. If the alert has additional parameters that influence when it activates, these will be described using the letters below:

- P = Alert is either disabled or different for pediatric patients. This does not include alerts that use weight, BMI, or height to scale for each patient.
- T = Alert uses trending, multiple values, medians, or averaging of values over time.
- F = Alert uses a formula or logic to calculate clinical parameters.

### Priority

Based on ISO’s definition of the potential result of failure to respond, and the onset of harm, AlertWatch has defined the priority of the alerts. In particular, they have different Priority levels that distinguish the urgency of the alert, along with characteristics that that they recommend to ensure the user of the medical device correctly interprets and responds to the most urgent situations. There are, in order of lowest to highest priority:

- I = Information Signals
- L = Low Priority
- M = Medium Priority
- H = High Priority

### Onset of harm

This ISO classification for onset of harm includes the following parameters. AlertWatch has added a None categorization for alerts that do not fall within the three ISO categories.

- I = Immediate. Having the potential for the event to develop within a period of time not usually sufficient for manual corrective action.
- P = Prompt. Having the potential for the event to develop within a period of time usually sufficient for manual corrective action.
- D – Delayed. Having the potential for the event to develop within an unspecified time greater than that given under “prompt”.
- N = None

### Census view

**Fig. 1:**
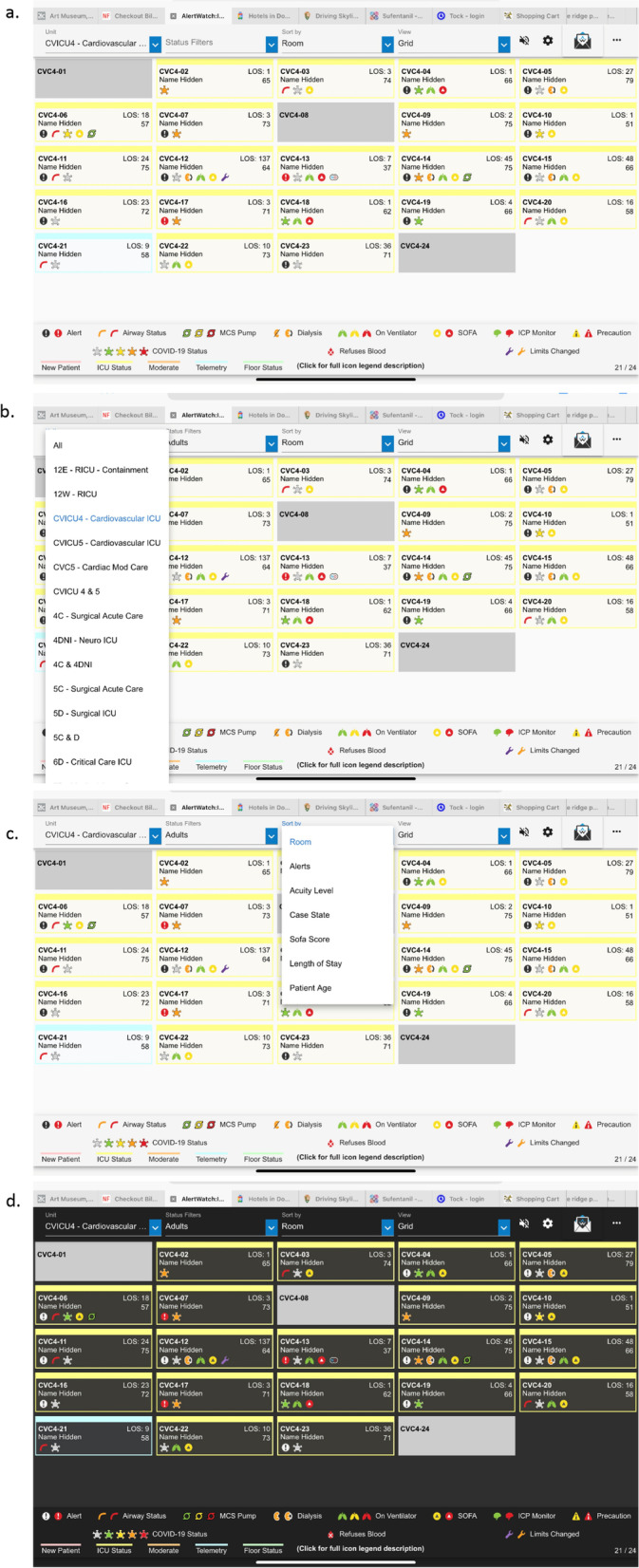
**a**. Census View: This is the census view of Alertwatch Acute Care (ICU and Floor). Each rectangle in the census represents a patient bed. Those colored gray are unoccupied beds. Each rectangle has icons which represent various aspects of patient care, for example, green lungs signifies a patient on mechanical ventilation. At the bottom of the census view are the various icons with their definitions. These are described in detail below and in Table 1. Each rectangle has the bed number, the patient’ name, length of stay and the age of the patient. **b**. Census View, Unit Selection: In the upper left of the census view there is a drop-down list so the individual units can be selected. **c**. Census View, Patient Type Selection: To the right of the drop-down list is another drop-down which allows selection of patients in order of acuity level, SOFA score and Case State. **d**. Dark Mode: This figure shows a dark mode view which can be initiated by tapping the sprocket, which is to the right of the ?, allowing the selection of the dark mode or light mode views

Figure 1a is a census view of an ICU. Each rectangle represents a patient and has the bed number, the length of stay, the patient name, their age and the icons representing alerts and other important statuses of the patient, e.g. on a ventilator or dialysis; as described above, Table 1.

If the user is on the care team of any patients, the census can be limited to those patients with the My Patients filter. At the top of the census view there is a drop-down list which allows selection of different units within the hospital: ICUs, step-down units or floor beds, Fig. 1b. After a provider logs in for the first time and selects a unit, all subsequent log-ins by that provider will default to the units they last selected.

To the right of the unit selection is a set of drop-down Status Filters that defaults to My Patients as just noted. These are the patients in which the user is on the patients care team in the EHR. This drop-down can also select patients by different therapies e.g. receiving mechanical ventilation, on extracorporeal membrane oxygenation (ECMO), treated with nitric oxide, etc. Additionally, a variety of other patient censuses may be selected; Alert Level, Acuity Level, sequential organ failure assessment (SOFA) Score etc. [12]. Finally, to the right of that drop-down, there is an option to the View; Grid, List or Multi Patient view. This allows for different presentations or other ordering of the patients by activity of alerts, by the attending providing care or by the acuity of the patient, Fig. 1c. To the right of the grid is an alarm silence selection and a “?” which links to INSTRUCTIONS for use, standard COLOR LIMITS, ALERTS & ICONS definitions, PAGING and STANDARD and EMERGENCY references, Fig. 1c and Table 2.

At the far right are three dots “…” which allows switching to different AlertWatch applications, AWOR (operating room), AWOB (obstetrics), or AWPACU (Post Anesthesia Care Unit).

Each rectangle represents a patient as described in the legend above, Fig. 1. At the bottom of the census there is a brief description of each of the icons by name and a description of the color coding for different levels of care of the patient in the unit, Fig. 1a and 1c. These colors indicate, a newly admitted patient, ICU status patient, a moderate care or floor status. Each of the icons are listed in Table 1 and will be described in more detail below. Each one has color coding, which designates the acuity of each of these icons, see Table 1. Table 1 also provides a list of all the alerts. In general, green is normal range, yellow is slightly abnormal, and red is abnormal, Table 2. The alerts come in three levels; a black alert is information of interest and is represented by a black circle, a more concerning alert will be in red, the most concerning alert will be flashing red and can be associated with a text notification to a care provider via a mobile device, Table 1.

### Patient view

**Fig. 2:**
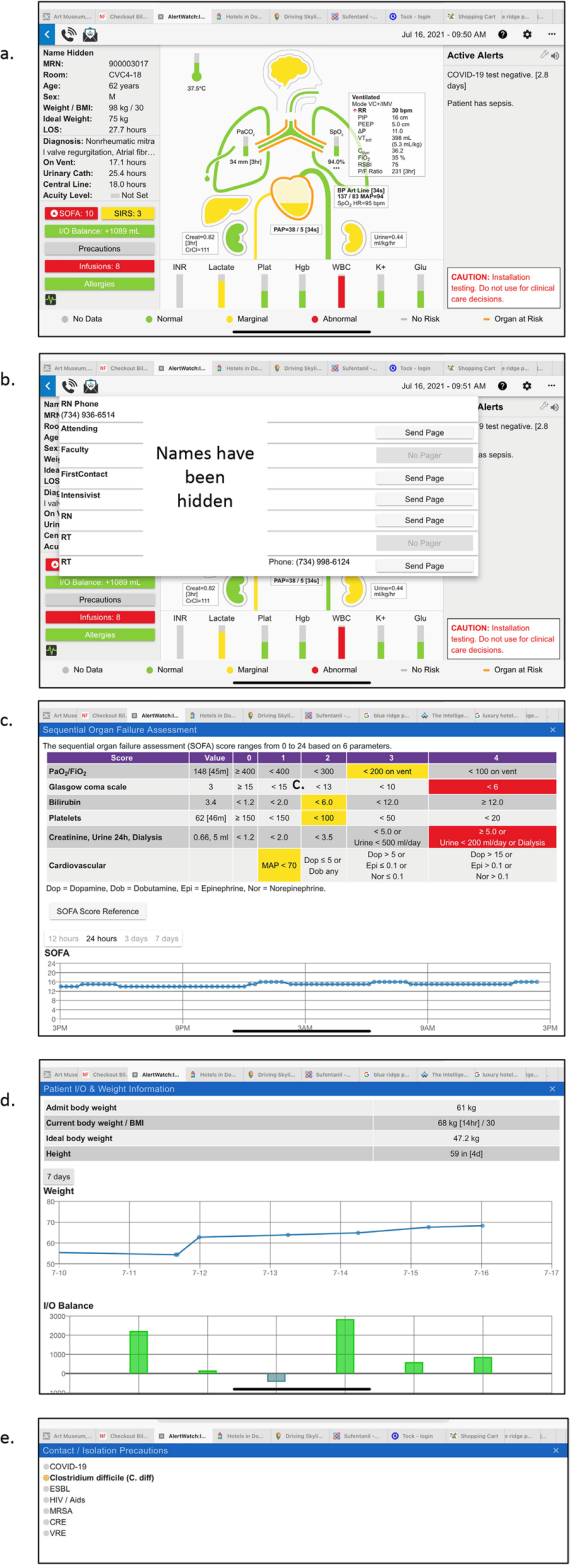
**a**. Patient View: When any patient is tapped in the census view, the patient view will appear, seen below. The patient view has three sections: on the right side are patient demographics and other patient information, which is described in detail in the text; in the center is the organ system icon view and to the right are the current active alerts. Below the patient are the current labs. The color coding of the display signifies within the normal range: (green), yellow: (slightly out of the normal range), and red: (being abnormal values). When any of the colored icons or squares are tapped the details of that aspect of the patient will be presented with trend plots of the relevant variables. The text alerts come in three severities: black alerts are informational, red text alerts are more important and red scrolling alerts are most important and may be programmed to automatically send a page to a provider. **b**. Patient View, Care Team Contacts: In the upper left of the patient view is an icon of a telephone. When it is tapped the patient’s care team and their contact information is presented in the drop-down menu. When viewing from a smart phone, tapping the telephone number will directly call the provider. **c**. Patient View Demographics: On the left-side of the patient view below the demographics are important variables such as ventilator days, urinary catheter days, etc. Below that are the four service scores and deterioration index. If the SOFA score is tapped, the table appears which determines which components of the SOFA score are in the normal and abnormal ranges in a trend spot of the SOFA score. **d**. Patient View, Information: Following the SIRS scores is an I/O box, it shows the trend of input and output fluids and are presented along with the patient’s weight and positive fluid balance for a 24-h period. Represented by a column above the zero-line (green) and a negative fluid balance (blue) for the 24-h is a column below zero-line. **e**. Patient View, Precautions & Infusions: Below the I/O balance are precautions, which note whether the patient has contact precautions and tapping that square will show which infectious agents are of concern. Below that are infusions. When the infusion is tapped the current infusions with their dosing rates are presented

Clicking upon a patient descriptor in the Census View opens a separate patient view in the same screen, Fig. 2a. The patient view has three panels. On the left is patient demographics and information, the middle section provides an icon view of the patient’s major organ systems with a beating heart and ventilating lungs. To the right is the Active Alerts Panel, which will list text descriptions of the active alerts for this patient. Alerts are color coded to prioritize severity: informational alerts are black text and represented by a black circle icon, for more important alerts the text is red (red alert icon), and the highest level of severity alerts are represented by a flashing circle icon and a scrolling red text alert and may be configured to activate a page to the appropriate care provider, as noted above. At the top left of the patient view there is a phone icon which provides contact information either by phone number or paging/text numbers for all the individuals on the patient’s care team, Fig. 2b. To the right of the phone icon is an envelope icon which allows users to directly contact the AlertWatch support team.

As noted in Fig. 2a, to the left-side of the Patient View screen shows the patients name, registration number, room location, age/gender, weight/BMI, Ideal Weight, and length of stay. The mid-portion shows the admitting diagnosis, below that ventilator days, urinary catheter days, central-line days, and acuity level; if an acuity level has been set by the care team. Below that are the sequential organ failure assessment (SOFA) and systemic inflammatory response syndrome (SIRS) scores, which are automatically calculated [12]. If these squares are tapped the window will provide the components of the scores and trends. For example, the SOFA table and trend plot is shown in Fig. 2c. Below the SIRS box is fluids inputs and outputs (I/O) balance. Hitting the I/O box will give a trend of the patient’s weight and the trend of I/O balance, Fig. 2d. The I/O balance is determined every hour and the cumulative plus or minus will be showed in the trend box. These data are extracted from the nursing flowsheet.

Below the I/O box is a display element containing isolation status. This signifies the level (if any) of isolation precautions required for this patient. If it is green there are none, if yellow there are standard isolation precautions, e.g. MRSA or C-difficile. If it is red, it signifies special precautions i.e. COVID-19. Selecting the isolation status icon it shows the specific infectious precaution. At the bottom, current infusion medications and allergies, Fig. 2e. As with all other icons and boxes when tapped, a pop-up window provides the specifics, e.g. allergies and infusion medications and doses of those medications.

**Fig. 3:**
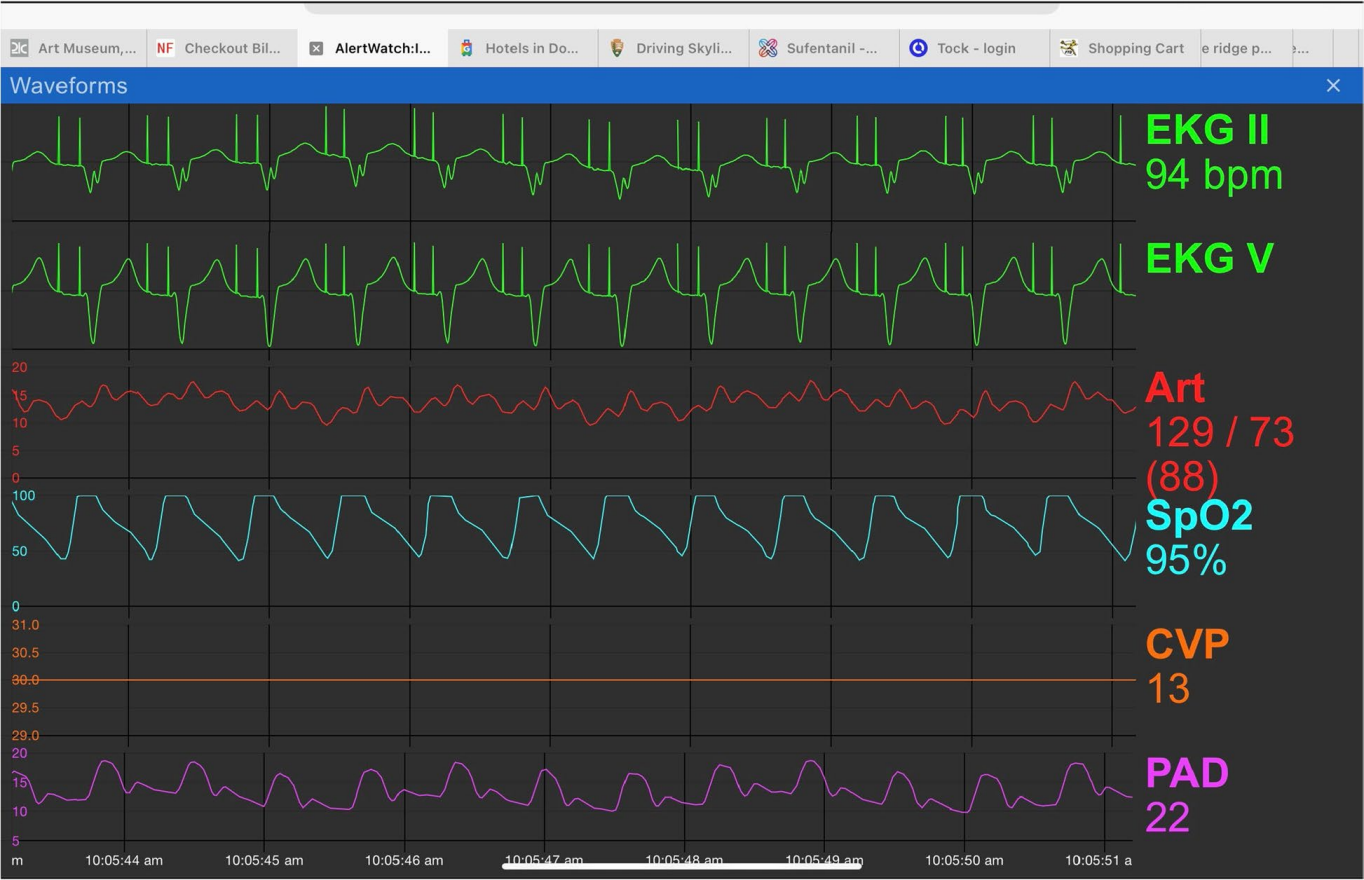
Physiologic Waveforms: If configured and available, when the icon in the lower left is tapped live physiologic waveforms will be presented

The icon at the lower left provides a link to the live waveforms the patient’s physiologic monitors, if they are on the hospital network, Fig. 3. At the very bottom of the patient view is the color coding for the icon-based system. Gray meaning no data are available for that aspect of patient, green is normal range, yellow is marginal and, red signifies abnormal values, Table 2. An orange outline signifies a co-morbidity associated with that organ system or lab, “At Risk.” For example, if the patient has diabetes there will be an orange outline surrounding the glucose lab measurement, Fig. 2a. Clicking on a graphic with an orange outline will give more information on the specific co-morbidity.

**Fig. 4:**
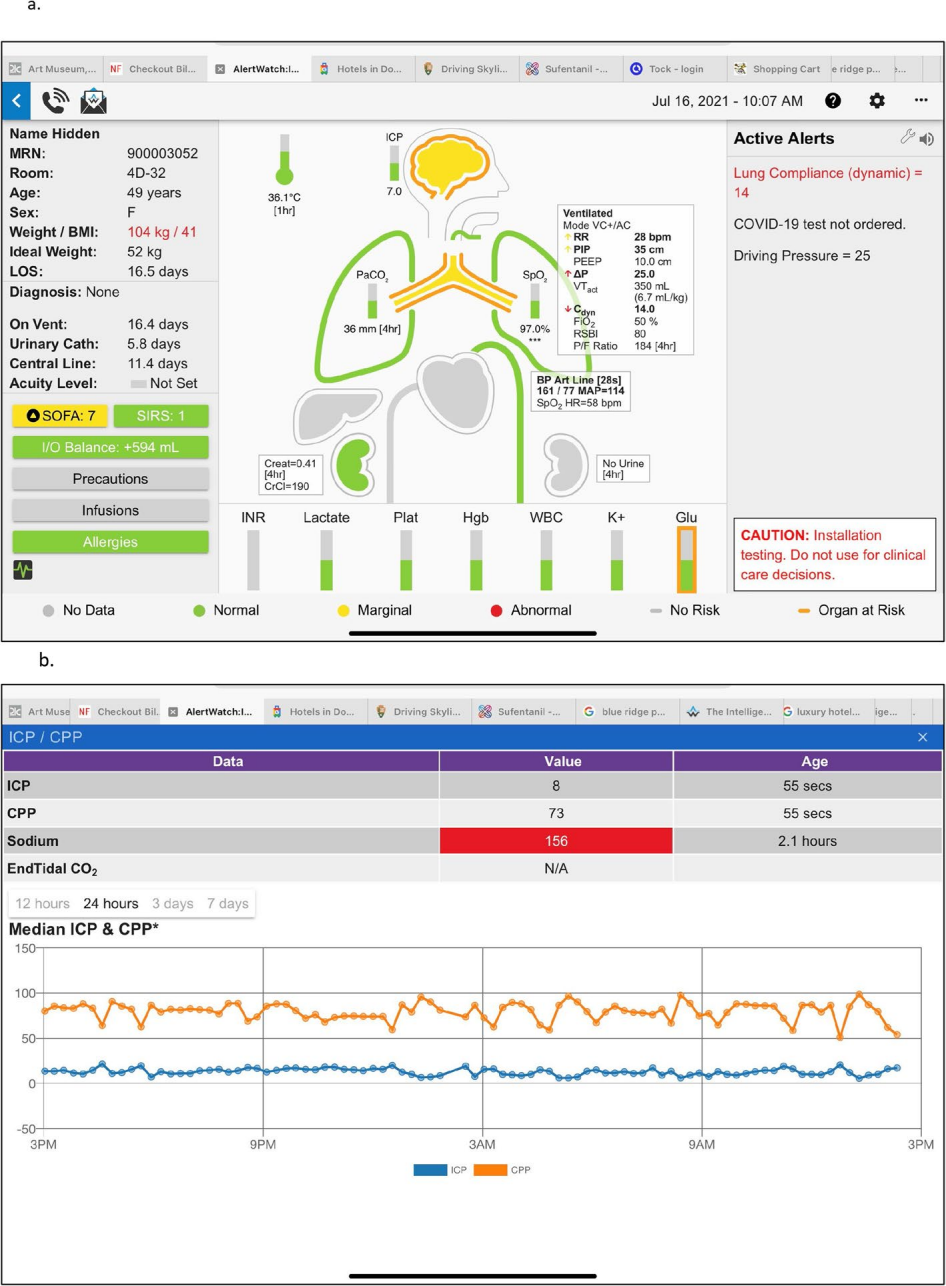
**a**. Patient View, Brain Information: The icons at the top of the patient view are the patient’s temperature and the brain. If the patient has an intra-cerebral pressure monitor a column will appear to the left of the brain. It will note intracranial pressure with normal and abnormal ranges by color coding. If the brain is outlined in orange the patient has a comorbidity such as stroke. The interior color of the brain is green if the mental status is normal and yellow if they have an abnormal CAM or RASs score. **b**. Patient View, ICP: When the ICP icon is tapped the intracranial pressure and the cerebral perfusion pressure (CPP) trend will be viewed

**Fig. 5:**
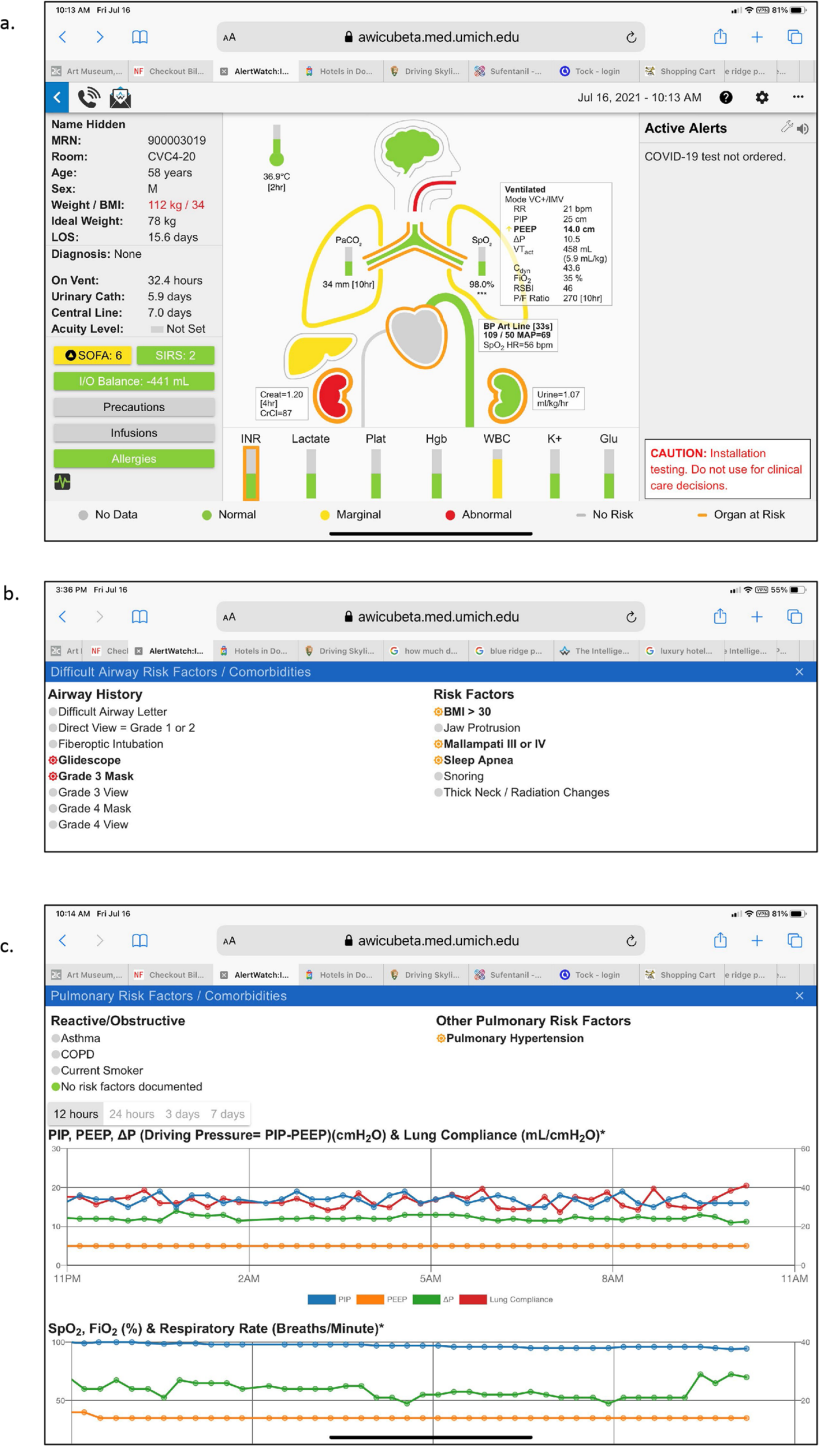
**a**. Patient View, Airway: Below the brain is an icon of an endotracheal tube (ETT). A green ETT means the most recent intubation was not difficult (Fig. 2a), if red it was difficult, below. If the ETT is orange, it means the airway exam had risk factors for possible airway difficulty. **b**. Patient View, Airway Risk Factors: When the ETT is tapped the specifics of the risk factors and experience for the previous intubation are presented. **c**. Patient View, Pulmonary Status: When the lung icons are tapped trend plots of oxygen saturation and PaO2/ FiO2 ratio are presented along with the most recent blood gas data

The center section of the patient view provides an anatomically organized icon-based view of the patient’s major organ systems, below this are the current labs. If there are special monitors or devices those icons will be present, automatically added. For example, an intracranial pressure (ICP) column/monitor will appear to the left of the head icon if present, Fig. 4a. The brain color is gray if there are no information regarding level of consciousness, green if the level of consciousness is normal and yellow if there is an abnormal confusion assessment method (CAM) or Richmond agitation-sedation scale (RAS) score [13, 14]. In all cases when the icon or lab is tapped a pop-up window will open giving the trend values of that parameter. For example, Fig. 4b shows the trends of ICP and cerebral perfusion pressure (CPP) in this patient with an intracranial pressure monitor. Below the brain is an icon of the endotracheal tube (ETT). If the ETT is gray there are no data regarding risk factors or history for intubation. If the ETT is orange it notes there are risk factors for intubation from the most recent airway exam, green if there is a history of easy masking and intubation and red if there is a history of difficult intubation, e.g. requiring fiber optic or video laryngoscope, Figs. 2a, 5a and b. If there is an icon of a tube below the jaw coming out of the neck it means the patient has a tracheostomy or a stoma, (Fig. 7a discussed later). Below the neck is the trachea and main-stem bronchi and lungs. If there are co-morbidities with the lungs, such as asthma, there will be an orange outline of the trach and main-stem bronchi, Fig. 4a. Each lung has data regarding oxygenation, SpO2 and ventilation, end expired carbon dioxide (ETCO2). If the patient is being mechanically ventilated, a ventilator box will be to the right of the ETT, noting the mode of mechanical ventilation: respiratory rate (RR), peek inspiratory pressure (PIP), positive end expiratory pressure (PEEP), Driving Pressure (PIP-PEEP), tidal volume, Dynamic Compliance, FiO2, rapid shallow breathing index (RSBI) and arterial oxygen partial pressure to inspired fraction of oxygen ratio (P/F ratio), Fig. 2a [15–17]. If this ventilator box is tapped there are trends of these parameters. If the lungs are tapped a window will show trending of SpO2 and ETCO2 as well as recent blood gas measurements, Fig. 5c.

**Fig. 6:**
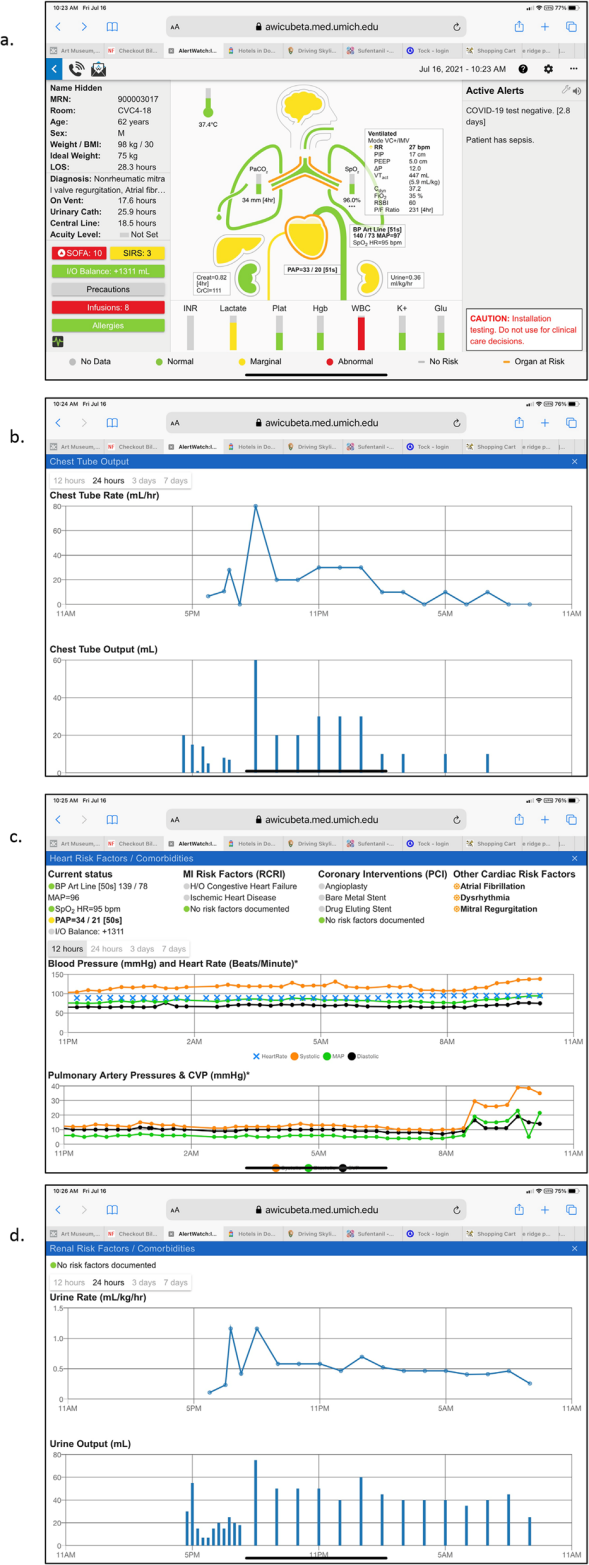
**a**. Patient View, Chest Tube: If the patient has a chest tube that will be noted coming from the lung on the left-side of the patient view. If the chest tube is green, the chest tube drainage is low; if the chest tube is red, the drainage is high (greater than 300 ml/hr., this is configurable). **b**. Patient View, Chest Tube Output: When the chest tube icon is tapped a trend plot of chest tube drainage and incremental chest tube output is presented. **c**. Patient View, Hemodynamics: The current blood pressure is presented to the right of the aortic arch icon and the current CVP or PAD pressures are presented below the heart. When the heart icon is tapped trend plots of blood pressure, pulmonary artery pressures and CVP pressures are presented. The patient’s cardiac comorbidities are also presented above the trend plots. **d**. Patient View,  Renal Function: By tapping the kidney on the right the trend and increments of urine output are presented

If the patient has a chest tube that icon will be in the lung on the left and the color of the chest tube will change as the chest tube output increases, Fig. 6a. When the chest tube is selected, the amount drained is shown as both increments and an hourly rate over time. If output exceeds more than 200 ml in one hour or 300 ml in 3 h, an alert is activated, Fig. 6b (these are configurable to site or patient specific needs).

Below the lung icon is the heart with aortic arch and inferior vena cava (IVC). The “fluid” level of the heart will be in the middle green, high yellow, high red or low yellow, low red depending on measurements of central venous pressure (CVP) or pulmonary artery diastolic pressure (PAD), if available and prioritized in a hierarchical manner. The green, yellow and red ranges of all values are in Table 2. If the heart has an orange outline it means their comorbidities, which can be determined by selecting the heart, will be presented along with blood pressure and CVP or PAP trends, Fig. 6c. If the patient has an implantable cardiac defibrillator (ICD) or a pacer these icons will attached to the Heart, Fig. 6a.

**Fig. 7:**
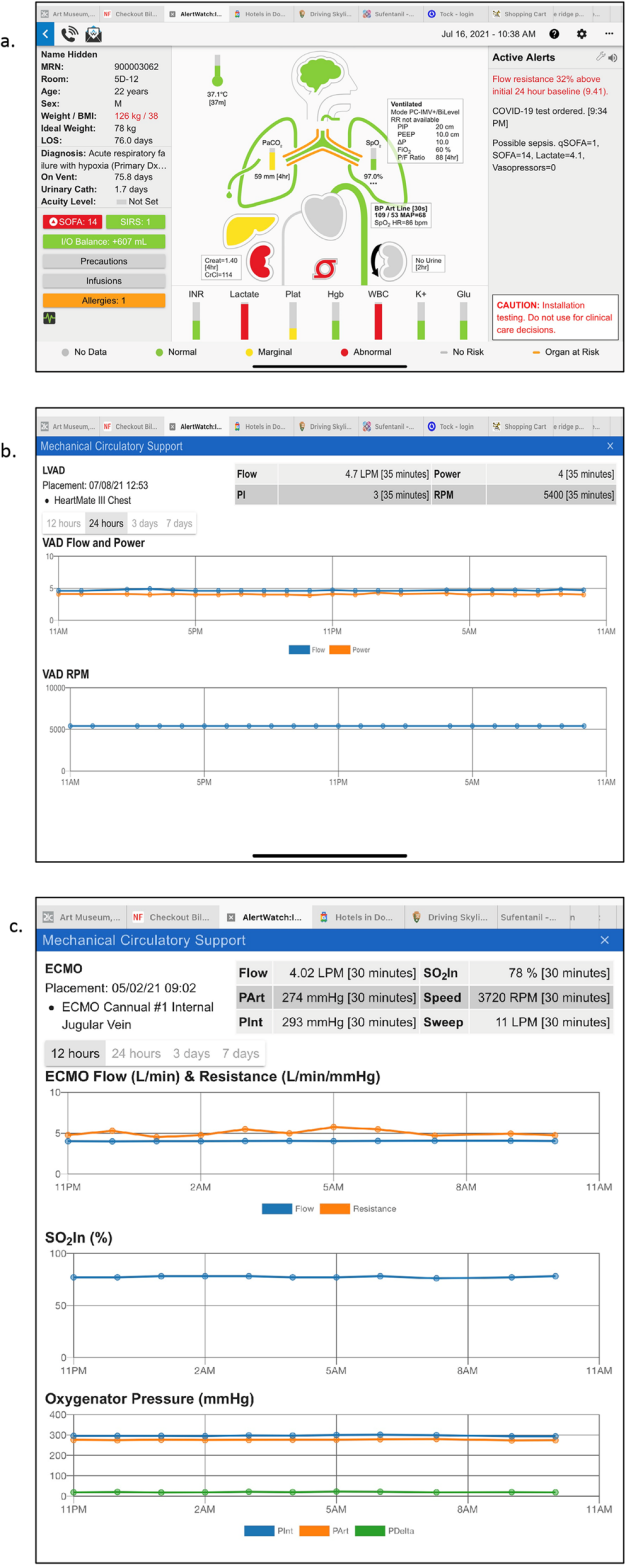
**a**. Patient View, Mechanical Circulatory Support: If the patient has mechanical circulatory support; ECMO (red), intra-aortic balloon pump (yellow), LVAD (green) icon is displayed below the heart. **b**. Patient View, LVAD: When the LVAD icon is tapped the flows, RPM and power trends are presented. **c**. Patient View, ECMO: When the ECMO icon is tapped the ECMO flow pressures, oxygenation saturation and resistance trends are presented

To the left of the heart is the liver, which will be green if liver function tests are normal and yellow if they are abnormal. As with all organ icons, if there is a disease such as cirrhosis of the liver, the organ will be outlined in orange. To the right of the heart is the current blood pressure and heart rate and if that box is tapped the trend of those values will be provided, Fig. 6c. The aortic arch will change colors from green to yellow to red depending on the blood pressure alert limits set by the system. On either side below the heart are the right and left kidneys. The kidney on the left-side provides an alert for chronic renal function as determined by the most recent creatinine value. If it is green the creatinine is normal, yellow if it is marginally high and red if the creatinine value is high, Table 2. Selecting the left kidney will provide trends of the creatinine as well as trends of blood urea nitrogen. The right kidney color designates the current values for urine output. Again, their range is a green, yellow and red depending on the limits set, Table 2. The urine output trend is provided by tapping that kidney, Fig. 6d. A curved arrow icon around a graphic representing the kidney represents a patient receiving continuous renal replacement therapy, Fig. 7a. A line through the kidney indicates a patient requiring hemodialysis, see icons and definitions, Table 1.

Below the icon to the human body are laboratory values for international normalized ratio (INR), lactate, platelets, hematocrit, hemoglobin, white blood count, potassium and glucose. As with all other values, tapping each column will provide a trend of those values and the colors of the column will depend on whether they are in normal, marginal or abnormal ranges.

On the right side of the patient view is the alert section where current active alerts will be presented either in black text, red text or scrolling red text depending on the urgency and importance of the alert. To the right of the alert section is a wrench icon which allows patient specific alerts to be configured.

#### Icons

A series of icons which are presented on the Census View and the Patient View that signify important aspects of patient status. These icons are defined at the bottom of the census view and in the online user guide and in Table 1. They are present on each patient square in the census view for the patients to which they apply.

### Alerts icon

As stated above, the alerts icon are circles with a “!” in the middle. The alerts are either black, red or flashing red noting the degree of importance for that alert, Table 1. These alerts are configurable at the institutional installation. Some icons which are present on the census view are also presented in the upper left of the patient view because they do not relate to a specific organ; COVID-19 status (see below) and Patient Refuses Blood Products, Table 1.

### Airway status

Again as noted above, the ETT icon can be presented in various colors; gray if there is no information, orange if there are risk factors for difficult intubation, green if there is a history of an easy masking and intubation and red if there is a history of a difficult intubation, Fig. 5a [18, 19].

### COVID-19 icon

This icon presents in five levels/colors; gray represents no data available (as it does for all aspects of AW), yellow means the test is pending, red is COVID-19 positive, green is COVID-19 negative or Antibody positive and orange if the COVID negative test result is greater than 4 days old, indicating potential need for re-testing (this time window is configurable based on site policies/procedures), Tables 1, 2 and Figs. 5a, 6a.

### Pump icon to signify mechanical circulatory support

The mechanical circulatory support (MCS) icon looks like a pump below the heart which can either be green for a left ventricular assist device (LVAD), orange for an intra-aortic balloon pump (IABP), and red for extracorporeal membrane oxygenator (ECMO), Fig. 7a. When these icons are tapped a window opens which provides specific data regarding that device. For example, Fig. 7b presents a patient with an LVAD device and the window presents flow, power, and RPM data as well as trends for that information. If the patient is on an ECMO device, the trend data will provide information regarding the flow, pressures, saturation, RPM and the sweeps speed. Trends are also provided for ECMO flow and resistance, as well as, venous and arterial oxygen saturation and oxygenator pressures, Fig. 7c.

### Dialysis

The kidney dialysis icon is orange with a slash through it if the patient is in complete renal failure and on hemodialysis. If it is an orange kidney with an arrow the patient is on temporary support with continuous renal replacement therapy (CRRT) as described above, Fig. 7a.

### Ventilator

The ventilator icon (lungs) is displayed on the census view when the patient is on a ventilator. The icon can be green, yellow or red depending on whether the ventilator pressures are in the normal, marginal or very abnormal range, e.g. high PIP or PEEP pressures as noted above, Figs. 2a, 7a. These alert color change limits are configurable. There is also a Ventilator Associated Event (VAE) alert, which will be discussed below in the Ventilator Management Tracking section. The VAE box is yellow if the FiO2 or PEEP have been increased to meet the criteria of a VAE; if these changes continue past midnight and red when midnight is passed [20, 21].

### Nitric oxide

If the patient is receiving therapy with nitric oxide (NO) an icon is shown in the upper right and the nitric oxide dose in parts per million is presented in the ventilator box. There is also a red alert for nitric oxide, which provides the duration and dose in the Alert section.

### SOFA

The SOFA score is in the left panel of the patient information button and also a red arrow icon. When the SOFA square is touched a box opens which provides the values which determine the SOFA score and also a trend, Fig. 2c. SIRS scores and trends are also displayed [12].

### ICP monitor

The brain icon is presented when the patient has an inter-cerebral monitoring catheter. This can either be intracerebral or a lumbar catheter used in vascular procedures. If the icon is green it means the ICP is in the normal range, if it is red it is an abnormal range. When there is a catheter measuring cerebral fluid pressure a bar column is displayed to the left of the brain with the current ICP pressure. Tapping the icon provides trends of ICP and the cerebral perfusion pressure (CPP) data, Fig. 4a.

### Other icons

There are other miscellaneous icons. There is an icon if the patient refuses blood products. If individual alert limits want to be changed for an individual patient, there is a wrench icon. If the wrench is purple it means a specific alert limit has been changed for this patient.

### Ventilator management tracking; TV and VAE

The Center for Disease Control requires measurement of Ventilator Associated Events (VAEs) in patients receiving mechanical ventilation [21]. Occurrence of a VAE represents a deleterious change in oxygenation: either by FiO2 increased by 10% or greater or an increase in PEEP by 3 cm H2O or greater over a previously determined 48-h baseline value. These events are said to have occurred if these changes continued past midnight [21]. Given their role in quality measurement and assessment of health system practice, it is important that providers reassess the patient’s status to determine if these increases in ventilator support were truly required to maintain adequate oxygenation. For this reason, the VAE alert in AWAC is presented in two stages. When the triggered values are exceeded a yellow VAE alert is presented until midnight when the VAE now meets criteria for occurrence.

### Sepsis alerts: the role of complex multiparameter decision support

Timely sepsis diagnosis and management is critical to the care of patients in the acute care setting. Early diagnosis and prompt initiation of antibiotics are proven to improve patient outcomes [22]. In 2018, the Surviving Sepsis Guidelines were updated to include a revised 1 h bundle of care which included measuring a lactate and re-measuring if initial was > 2 mmol/L, obtaining blood cultures prior to antibiotics, prompt administer broad spectrum antibiotics, and infusion of 30 ml/kg crystalloid for hypotension or lactate ≥ 4 mmol/L or initiation of vasopressors if hypotensive during or after fluid resuscitation [23]. Use of protocols to assist in early identification and treatment of sepsis have been shown to improve outcomes when utilized in the care of septic patients [24]. Decision support tools may have a role in identification and prompt management to improve patient care.

AWAC is designed to help providers both assess patients and provide alerts to remind them of various actions critical to the management of these patients and implement bundle-based recommendations. To facilitate adherence to the sepsis protocol, AWAC can alert/page the specific providers responsible for the various tasks: MDs for orders, RNs for blood draws and antibiotic administration and pharmacy for antibiotic preparation. If sepsis has been documented in the EHR, a black alert will appear or a red alert if septic shock is documented. Alerts for possible sepsis are based on the Third International Consensus Definitions for Sepsis and Septic Shock [25] and will appear if this diagnosis is present in the EHR or if the patient is ≥ 18 years old, qSOFA ≥ 2, SOFA score ≥ 2, and lactate is ≥ 2 mmol/L. If sepsis or septic shock is documented in the EHR, a window will be prompted with various actions that need to be performed within a given time window, Table 3.

**Table 3:** Organ Specific Sepsis Alerting

Alert/Column	Patients to check	Start of Window	End of Window	Logic
Blood Culture Status	All	T – 24 h	T + 3 h	Blood Culture procedure order within the window
Antibiotic Status	All	T – 24 h	T + 3 h	Antibiotics administration exist within window
First Lactate Status	All	T – 6 h	T + 3 h	Positive = Lactate result exists
Second Lactate Status	First Lactate result ≥ 2 mmol/L	T	T + 6 h	Positive = Lactate order or result within timestamp after initial result
Vasopressor Status	Septic Shock BPA only	Earlier of T or when SBP < 90	T + 6 h	Positive = Vasopressor administration within window

T = time that sepsis or septic shock was documented. SBP = systolic blood pressure

This alert text will include everything that is due and when. If a test is completed beyond the end of the window, that segment of the alert will be removed. The logic behind the alerts is consistent with known sepsis diagnosis and treatment guidelines [24, 25]. For lactate, AWAC will use the last lactate value measured within 6 h prior to documented sepsis or the first measured in the hours following a sepsis diagnosis. If the lactate is < 2 mmol/L or there is none, then no alert for a second lactate is generated; however, if one is present and is ≥ 2 mmol/L, AWAC will search for a lactate value within 6 h of that value and if not present, alert that one needs to be evaluated.

### Management of continuously monitored data: BP, SpO2, HR and RR

Patients in ICUs and step-down units are usually continuously monitored with networked physiologic monitors. Because of the need to address acute changes in these vital signs, alert limits are set to alarm when limits are breached. Unfortunately, artifacts occur, often due to motion, and resulting alarm frequency is so high alarm fatigue results [1–3]. To try to minimize the problem, AWAC employs scrolling median values to trigger alerts. The median values of each variable is determined over a 5-min period which is updated every one minute. The scrolling median time period is configurable. This method will remove short term “outlier/artifact” values reducing these measurements producing an alarm, but will delay the alert by a few minutes depending on the scrolling period selected.

### Mobile use

AWAC has been configured and tested for use with mobile devices; tablets and smart phones. An application is available in the Apple App store by searching “Alertwatch Inc.” VPN access is required for out of hospital use. It opens to the census view and when a patient is selected, it is designed to swipe to view all the sections: patient view, patient information and alerts, provider contact and waveforms. The provider can be called directly if their phone numbers are in the EHR.

## Discussion

Automated decision support systems such as AWAC, may be utilized as back-up surveillance to support nursing care at the bedside or as part of a remote tele-ICU service. It has long been determined that even highly trained and motivated personnel’s ability to detect adverse events deteriorates over a matter of hours. This phenomenon was originally demonstrated in the context of assessment of the vigilance of sonar operators to detect enemy submarines [26]. The aviation industry has addressed this issue as the complexity of the aircraft’s monitoring systems increased. Information from multiple dials were integrated into a single multifunction display [4]. This primary flight display shows a horizon with few numbers but in the background processes multiple streams of information and provides alerts to the pilot in order of importance when concerning or dangerous situations occur. In aviation these systems have been implemented to reduce data overload causing the pilot to lose situational awareness. Broad implementation of this technology reduced the commercial crashes from one in a million flights to less than one in 16,000,000 flights despite increasing complexity of underlying systems [27]. Introduction of systems which summarize multiple sources of clinical information may support provider awareness of changing clinical status and support delivery of high complexity care.

Multifunction displays have been developed and deployed within routine anesthesia care [5, 7]. In a six-year retrospective study comparing users versus non-users of AWOR it was found that use of this system was associated with improved process of care compliance in management of blood pressure, tidal volume and fluid management [7]. Its use was also associated with a $3,500 decrease in patients encounter charges. Additional study has associated the use of alerting display with improved compliance with glucose management guidelines [28].

Further work has demonstrated utility of an automated monitoring system in Labor and Delivery (L&D) environments. Life threatening post-partum hemorrhage (PPH) is a rare event which necessitates immediate intervention to save the mother’s life [29]. PPH is associated with significant maternal mortality. The American College of Obstetrics and Gynecology (ACOG) and others have published guidelines for risk assessment and surveillance of mother; Maternal Early Warning System (MEWS) [29]. In an attempt to improve maternal surveillance another version of AlertWatch was developed for L&D; AWOB [6, 8]. In a recent observational study comparing AWOB to MEWS with the assumption that compliance with MEWS was 100%, AWOB had a better positive predictive value (PPV) for severe postpartum hemorrhage [8]. In addition, because AWOB retrieves vital signs directly from the monitoring network, AWOB detected nine severe cases of hemorrhage that MEWS did not detect [8]. An overview display mode used in the anesthesia, obstetrics and nursing workrooms allowed the simultaneous monitoring of multiple patients, perhaps in a manner more analogous to a control tower maintaining vigilance of multiple aircraft [30, 31]. AWOB has been well accepted by clinicians in Labor & Delivery with a majority of providers feeling the system should remain in use and that it improved patient safety [9].

This current manuscript describes an acute care focused version of AlertWatch which has similarities to prior operating room and labor and delivery specific versions. When considered in an ICU deployment, much like OR patients, the population is seriously ill and at high risk of further deterioration and therefore, have extensive monitoring with skilled providers – ICU Nurses. But additionally the patient’s primary nurse has multiple distracting tasks which requires them to leave the bedside. For this reason, the monitors have high/low alarm triggers meant to alert the nurse to come to the bedside, but have been well documented to produce alarm fatigue [32]. Furthermore, in ICU environments the provider team is required to manage multiple patients dispersed over a geographic unit. The role of remote surveillance and communication systems and services is well established in Critical Care [33]. These systems mostly rely on consultation by request and/or surveillance by another layer of provider viewing the EHR and monitors [33, 34]. Which again relies on human vigilance.

A preliminary version of AWAC was developed and implemented in 2017 to monitor floor patients at a hospital by alerting a surveillance consultant at another hospital [10]. Safavi et al. studied the feasibility and utility of alerting for 6 physiologic and lab values for a 24 bed surgical floor. The nearly 1.6 million vital sign and labs electronically reviewed resulted in 2.6 alerts pre week (0.3 per shift), 88% of which were actionable and 68% resulted a in change in patient management [10]. They concluded that electronic remote surveillance can provide actionable alerts without alarm fatigue.

The AWAC system may have some advantages in that it can automatically send notifications to specific providers; RN, RT, MD depending the management protocols without depending on human vigilance. As noted above, AWAC is not limited to the ICU. The system can be applied to step-down beds, floor units and even the Emergency Department [10]. The data feeds are the same for every patient in the hospital. If there are no data available for a specific field, that organ is just gray.

The impact of AWAC on ICU patient care remains to be determined. Improvement needs to be demonstrated in both process of care, patient outcomes and acceptability by providers. Implementation of systems like AWAC may help expand to opportunities for remote surveillance, increased monitoring and advance individualized care plans. Para-EHR systems which aggregate information may offer opportunities for deploying sophisticated care algorithms derived from artificial intelligence (AI). Douville et al. recently employed AI techniques to predict the need for mechanical ventilation in COVID-19 patients [35]. They found that the calculated variable of SpO2/Estimated FiO2 (non-intubated patients) was the most predictive and could be continuously determined with SpO2 monitoring.

### Conclusion

Lessons learned from the aviation industry may offer opportunities for improvement in medical care—Checklists, crew/team resource management, the multifunction display and the flight tower have led to substantial improvements in aviation safety. This paper describes a system which retrieves, integrates, analyses and displays medical data and alerts providers to possible issues regarding the patient’s condition. Studies utilizing AWAC are required to determine its effectiveness in improving safety and quality of patient care.

## Availability and requirements

Project Name: AlertWatch AC.

Project Home Page: www.AlertWatch.com

Operating System: Platform independent.

Programming Language: MS-SQL, C#.NET with Java script front-end.

Other Requirements: Javascript enabled web browser.

License: No license required for viewing demo (available online at: http://www.alertwatch.com). FDA cleared software medical device requires licensing for installation.

Any restrictions to use by non-academics: No.

## Data Availability

This is proprietary software for this FDA cleared medical device. We are unable to provide the software, which is in excess of 10,000 lines of code. There is no repository as this software does not store data.

## Abbreviations

ACOG: American College of Obstetrics and Gynecology
AI: Artificial intelligence
AWAC: AlertWatch Acute Care
AWOR: AlertWatch Operating Room
AWOB: AlertWatch Labor & Delivery
AWPACU: AlertWatch Post Anesthesia Care Unite
CAM: Confusion assessment method
CPP: Cerebral perfusion pressure
CRRT: Continuous renal replacement therapy
CVP: Central venous pressure
ECMO: Extracorporeal membrane oxygenation
EHR: Electronic health record
ETCO2: End expired carbon dioxide
ETT: Endotracheal tube
I&O: Intake and output
IABP: Intra-aortic balloon pump
ICU: Intensive care unit
ICD: Implantable cardioverter defibrillator
ICD-10: International Classification of Disease, Tenth Revision
ICP: Intracranial pressure
INR: International normalized ratio
ISO: International Organization for Standardization
IVC: Inferior vena cava
LVAD: Left ventricular assist device
MEWS: Maternal early warning system
MCS: Mechanical circulatory support
NO: Nitric oxide
OR: Operating room
PAD: Pulmonary artery diastolic pressure
PEEP: Positive end expiratory pressure
P/F Ratio: Partial pressure to inspired fraction of oxygen ratio
PIP: Peak inspiratory pressure
PIP-PEEP: Driving pressure
PPH: Post-partum hemorrhage
PPV: Positive predictive value
RAS: Richmond agitation-sedation scale
RPM: Revolution per minute
RR: Respiratory rate
RSBI: Rapid shallow breathing index
SBP: Systolic blood pressure
SIRS: Systemic inflammatory response syndrome
SPV: Systolic pressure variation
SOFA: Sequential organ failure assessment
SVV: Stroke volume variation
TV: Tidal volume
VAE: Ventilator associated event
VPN: Virtual private network
